# Highly reproducible rat arterial injury model of neointimal hyperplasia

**DOI:** 10.1371/journal.pone.0290342

**Published:** 2023-08-17

**Authors:** Richard P. Tan, Jui Chien Hung, Alex H. P. Chan, Angus J. Grant, Matthew J. Moore, Yuen Ting Lam, Praveesuda Michael, Steven G. Wise

**Affiliations:** 1 Faculty of Health and Medicine, School of Medical Sciences, University of Sydney, Sydney, NSW, Australia; 2 Charles Perkins Centre, University of Sydney, Sydney, NSW, Australia; INSERM, Université de Bordeaux, FRANCE

## Abstract

Models of arterial injury in rodents have been invaluable to our current understanding of vessel restenosis and play a continuing role in the development of endovascular interventions for cardiovascular disease. Mechanical distention of the vessel wall and denudation of the vessel endothelium are the two major modes of vessel injury observed in most clinical pathologies and are critical to the reproducible modelling of progressive neointimal hyperplasia. The current models which have dominated this research area are the mouse wire carotid or femoral injury and the rat carotid balloon injury. While these elicit simultaneous distension of the vessel wall and denudation of the luminal endothelium, each model carries limitations that need to be addressed using a complementary injury model. Wire injuries in mice are highly technical and procedurally challenging due to small vessel diameters, while rat balloon injuries require permanent blood vessel ligation and disruption of native blood flow. Complementary models of vascular injury with reproducibility, convenience, and increased physiological relevance to the pathophysiology of endovascular injury would allow for improved studies of neointimal hyperplasia in both basic and translational research. In this study, we developed a new surgical model that elicits vessel distention and endothelial denudation injury using sequential steps using microforceps and a standard needle catheter inserted via arteriotomy into a rat common carotid artery, without requiring permanent ligation of branching arteries. After 2 weeks post-injury this model elicits highly reproducible neointimal hyperplasia and rates of re-endothelialisation similar to current wire and balloon injury models. Furthermore, evaluation of the smooth muscle cell phenotype profile, inflammatory response and extracellular matrix within the developing neointima, showed that our model replicated the vessel remodelling outcomes critical to restenosis and those becoming increasingly focused upon in the development of new anti-restenosis therapies.

## Introduction

Minimally invasive endovascular device intervention has revolutionised the treatment of cardiovascular diseases, due to high acute success rates and low periprocedural complications [1,2]. Despite their success, the most prevalent mode of failure is the ensuing tissue remodelling responses which cause the vessel to re-narrow over the mid- to long-term in a process clinically termed restenosis. The frequency and extent of restenosis has been significantly reduced following the advent of balloons and stents eluting powerful cytotoxic drugs such as sirolimus and paclitaxel [3,4]. These agents act non-specifically on vascular cells to prevent vessel re-narrowing but do not act on the underlying drivers such as chronic inflammation [5]. In coronary applications, early iterations of drug-eluting stents healed poorly and increased the risk of clotting [6,7], while modern devices deployed in the peripheral circulation are only effective for the duration of drug elution [8]. Continued refinement of drug-eluting technology and exploration of new pharmacological pathways to combat restenosis requires appropriate models of these biological processes. A heavily relied upon tool that has been fundamental to our current understanding are injury-based animal models that enable high-throughput evaluation of the cellular and molecular responses that drive restenosis, including endothelial cell dysfunction, vascular smooth muscle cell (SMC) phenotype switching, and formation of occlusive neointimal hyperplasia (NH) [9].

High pressure balloon catheter inflation characteristic of either stent or drug-eluting balloon delivery causes a significant vessel injury which leads to mechanical distention of the vessel wall and disruption of the native, protective endothelium [10]. Subsequent SMC proliferation and migration from the vessel wall into the lumen is the characteristic hallmark of NH, resulting in wall thickening and gradual loss of vessel patency [11]. Local inflammatory mediators provide the stimuli for SMC migration in a continuum of acute and chronic inflammatory reactions orchestrated by innate immune cells such as pro-inflammatory M1 macrophages. Secreting myriad cytokines and chemokines including IL-1β [12] and TNF-α [13], macrophages drive SMC differentiation from their native ‘contractile’ phenotype expressing PCNA to a highly proliferative ‘synthetic’ form with elevated SMC-α expression. Synthetic SMCs comprise the bulk cellular components of the occlusive neointima in addition to laying down a dense matrix rich in proteoglycans, which contribute physically to increased intimal thickness and supports further SMC migration and proliferation in addition to immune cell adhesion [14]. These mechanisms of NH development are commonly observed in current rodent-based vessel injury models, validating their use as practical and clinically significant. Using the carotid artery as a model system, several rodent-based approaches have been established and widely characterised to not only study vessel injury but also validate potential drug-elution therapeutics as a valuable “proof-of-concept” tool.

The two predominantly used models of mechanically-induced NH are the wire denudation and balloon injury models. Wire denudation utilizes an angiocatheter guide wire that is repeatedly inserted into a mouse carotid or femoral artery to simultaneously denude the endothelium and distend the vessel wall. Despite rapid NH development within 2 to 4 weeks, the key limitations are its technical difficulty and corresponding reliance on operator skill, due to the small size of mouse arteries and demonstrated by the high variability in restenosis levels reported across the literature [15,16]. Alternatively, some groups use the balloon model in rat arteries with larger vessel diameters for easier handling. In this model, a balloon catheter is inserted through a rat carotid artery and is repeatedly overexpanded and pulled along the vessel to similarly denude the endothelium and distend the vessel wall in a single procedure. SMC proliferation ensues and NH development typically peaks after 2 weeks [17,18]. Due to its improved surgical feasibility, balloon injury has been extensively used in the development of a wide range of anti-restenosis therapies including antibodies [19], recombinant proteins [20], gene therapy [21], and even the compounds used in current drug-eluting devices. However, while the size of the rat carotid allows for improved surgical handling relative to mouse arteries, the length of the required balloon catheter necessitates carotid artery lengths of at least 3 cm to reproducibly drag the balloon across the vessel length. This subsequently requires ligation of distal blood vessels past the internal-external bifurcation to maintain haemostasis, causing permanent disruption to native blood flow until the injury window is complete and the vessel harvested for analysis.

Development of a complementary injury model replicating the accessibility of the larger sized rat carotid vessel but using commonly available surgical equipment that allows for arterial injury without the permanent ligation of distal blood vessels would provide an invaluable expansion of current rodent models available for modelling NH. In this study, we report a novel carotid injury model using a surgical procedure the combines mechanical vessel distention and endothelial denudation into two distinct, sequential steps. This is achieved by combining the expansion of microforceps to a maximum width followed by the insertion of a needle catheter through an arteriotomy of the rat common carotid artery. Use of these tools allowed for greater control over the length of vessel injury requiring only 1.5 cm of the common carotid artery without additional permanent vessel ligation. Using male Sprague Dawley rats aged 7–10 weeks, we demonstrate that the microforceps and catheter injury is a simple microsurgical procedure that takes an operator roughly 20 minutes to complete from starting incision to final suture. Explanted rat carotids exhibit the classical vessel injury responses that include elevated M1 macrophage activation and pro-inflammatory cytokine secretion, SMC contractile-to-synthetic differentiation, and neointimal hyperplasia. Over multiple batches of surgeries, this model shows high reproducibility of these responses at a rate over 2 weeks that would allow deeper examination of pathological mechanisms and precise evaluation of restenosis therapies.

## Materials and methods

### Animals

Animal experiments were conducted in accordance with the Australian Code of Practice for the Care and Use of Animals for Scientific Purpose and issued under the Sydney Local Health District Animal Welfare Committee Ethics Protocol 2017/014. Male Sprague Dawley rats aged 7–10 weeks averaging 300 g were obtained from Animal Resources Centre (Perth, WA, Australia).

### Surgical procedure

#### Pre-operative

All surgical tools required in this procedure: fine pointed forceps, pointed forceps, spring scissors, and surgical scissors were sterilized using autoclave prior to surgery and maintained during surgery using a bead sterilizer (S1 Fig). Rats were placed in an anaesthetic induction chamber then filled with 5% (v/v) isofluorane in 100% oxygen at a flow rate of 1 L/min. Rats were left in the induction chamber until they were unresponsive to external stimuli. Depth of anaesthesia was confirmed using pedal and palpebral reflex, prior to removal from the induction chamber. A cocktail i.p. injection of ketamine (80 mg/kg) and medetomidine (0.5 mg/kg) was given to maintain anaesthesia for at least 45 minutes (surgical time should take no longer than 20 minutes) followed by pre-op fluid support (Ringer’s solution 10 ml/kg). Buprenorphine (0.05 mg/kg) was also delivered subcutaneously as an analgesic and ocular lubrication applied.

#### Injury & post-operative car

1. Anesthetised rats were placed in the supine position and using an electric shaver, hair was removed from the neck (Hair removal was performed at table separate from the operating table).
2. Rat were placed in the supine position over a heat mat (at 37°C) on the operating table, betadine applied followed by surgical drapes, leaving only the neck exposed.
3. Lignocaine anaesthetic (2 mg/kg, not exceeding 4 mg/kg per rat) was given subcutaneously prior to using a No.22 scalpel blade to make a superior-to-inferior 5cm incision on the side of the neck, starting roughly 3 cm below the jaw and 3 cm to the side of the neck midline. (Syringe will be drawn back before injection to ensure a blood vessel has not been entered)
4. Expose the underlying muscles (masseter and digastric), being careful with the trachea and gently push aside to expose the common carotid artery.
5. Secure two arterial clamps (S&T microsurgical instruments B-300401A) spaced around 1.5 cm apart at the proximal and distal ends to the exposed artery. Note: the top clamp is placed just below the bifurcation to the internal and external arteries, and the bottom clamp placed further proximal on the common carotid artery. This will occlude blood flow in the arterial segment which should last for only 15–20 minutes. Diagram included in S2 Fig.
6. Using spring scissors make a small entry incision at the proximal side of the carotid artery and flush with saline to remove the blood with sterile gauze from the artery.
7. Insert microforceps (World Precision Instruments 500373-T) into the small entry incision until they reach the distal clamped end of the artery.
8. Expand the microforceps to the maximum set width (1.55mm) and retract while expanded.
9. Repeat steps 8–9 four additional times.
10. Insert 22g catheter into the small entry incision until it reaches the distal clamped end of the artery, and pull the catheter out.
11. Repeat steps 10–11 four additional times.
12. Close the arteriotomy using a 9–0 silk suture using a single interrupted stitch and release distal and proximal clamps to restore blood flow.
13. Repeated steps 4–12 on the other side of the carotid artery for bilateral injury (optional).
14. Close the neck surgical incision by 3–0 silk suture using a continuous stitch.
15. To prevent infection, rats will be given an intramuscular injection of a prophylactic antibiotic; cephazolin (20 mg/kg) post-operatively prior to recovery from isoflurane.
16. To further support the animals during recovery, Ringers fluid (warmed to 37°C) will be subcutaneously injected at 10 mL/kg.
17. The animal will be allowed to recover on a heat mat (37°C) and monitored continuously until it has regained its righting reflexes.

#### Explant

1. Rats were anaesthetised using the described pre-operative procedure (section 2.2.1) prior to being placed in the supine position over a heat mat (at 37°C) on the operating table.
2. Using surgical scissors the neck incision was reopened and underlying muscles masseter and digastric moved aside and carotid artery exposed.
3. Using the arteriotomy suture as a landmark, place a vessel clamp just below/proximal to the suture and another spaced 1.5 cm apart distally (the space between the clamps will lie over the common carotid artery where the injury was performed). Cut the vessel between the two clamps to remove the segment and flush with heparin-saline immediately to prevent the blood clot.
4. Euthanise animal with a single I.V. bolus of pentobarbital (325mg/ml)

#### Histology

Explanted arteries were fixed in paraformaldehyde (PFA) (4%) overnight and dehydrated through an ethanol gradient. Samples were then embedded in molten paraffin before being cooled at room temperature to allow the paraffin to solidify. The entire length of the artery was cut into 5 μm thick cross-sectional slices using an electronic rotary microtome (ThermoFisher Scientific, HM 340E) and mounted onto microscope slides with three sections per slide. Equidistant slides were selected from across the entire length of the carotid to represent proximal, middle, and distal segments (S3 Fig). Paraffin sections were deparaffinised using xylene and rehydrated for histopathology stains including Haematoxylin and Eosin (H&E, Sigma, 51275 & R03040-74) for neointimal hyperplasia, Picro Sirius Red (PSR, Abcam, ab150681) for collagen, and Alcian Blue (Sigma, B8438) for proteoglycans using supplier-provided protocols. For immunohistochemistry staining, sections were stained with antibodies against CD31 (Abcam, ab28364, 1:200) for endothelialisation, SMCα (Abcam, ab5694, 1:200) and PCNA for smooth muscle cell contractile and synthetic phenotype respectively, and CD68 (Abcam, ab92552, 1:500), MHC Class II (Abcam, ab180779, 1:500), IL-1β (ThermoFisher, P420B, 1:200), and TNF-α (Abcam, ab6671, 1:200) for vascular inflammation. Secondary antibodies used were Goat Anti-Rabbit IgG conjugated AlexaFluor 594 (Abcam ab150080, 1:250) and Goat Anti-Rabbit IgG conjugated AlexaFluor 488 (Abcam ab150077, 1:250). Slides were coverslipped with mounting media with DAPI (Abcam, ab104139, Aqueous Fluoroshield). Secondary antibody control slides were performed to ensure specific binding of primary antibodies (S4 Fig).

### Image analysis

Analysis of histology and immunohistochemistry slides was done using ImageJ. For Haematoxylin and Eosin staining, neointima was quantified as percentage of total lumen area defined by the internal elastic lamina of the vessel wall. For PSR and Alcian Blue staining, proteoglycan and collagen deposition was quantified using the ‘Colour Threshold’ function in ImageJ to calculate amount of positive red or blue staining, respectively, present within the neointima and vessel wall. Fibrotic capsule was represented as total adventitial tissue area surrounding the graft. For SMCα, PCNA, CD68, MHC Class II, IL-1β, and TNF-α counts within the neointima was quantified using a constant threshold intensity and positive staining area measured. CD31 lumen coverage was quantified by first measuring the lumen circumference, followed by measuring the length of endothelium showing positive staining. Lumen coverage was then calculated as length of CD31+ staining divided by lumen circumference.

### Statistical analysis

Data are expressed as mean ± standard error of the mean (SEM) as calculated using GraphPad Prism 9 (Graphpad Software, San Diego, California) and statistically significant differences were determined by t-test with no corrections. Statistical analysis was performed on the averaged data comprising of all three vessel regions (proximal, middle, distal). The bar graphs in this paper represent the average amongst the vessels in each group with each vessel consisting of an average of its three regions (S3 Fig). p < 0.05 was considered statistically significant. *, **, and *** represent p < 0.05, p < 0.01, and p < 0.001, respectively.

## Results

### Surgical feasibility

**Fig 1:**
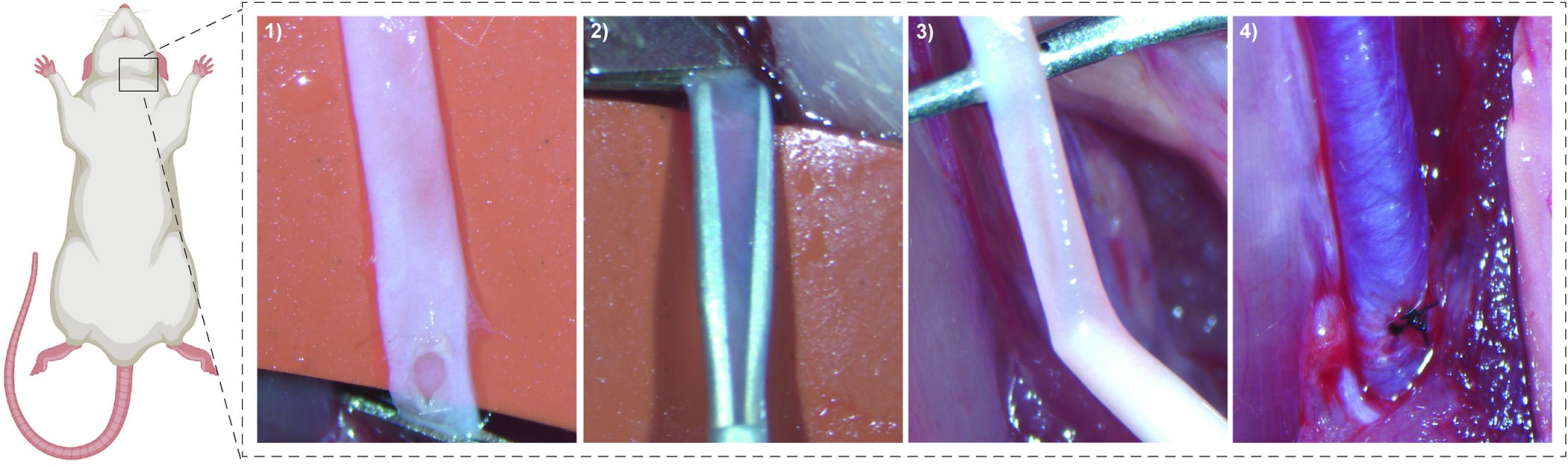
Surgical procedure of the combined microforcep and catheter rat injury model. Beginning with 1) arteriotomy of the common carotid artery 2) insertion and expansion of microforceps to mechanically distend the vessel wall, followed by 3) insertion of needle catheter to denuded the vessel endothelium after which 4) the arteriotomy is sutured closed and blood flow re-established. Rat schematic created with BioRender.com.

Under anaesthesia, the right common carotid artery was isolated and arteriotomy performed. Microforceps were inserted and expanded followed by catheter insertion, together repeated four times to mechanically distend the carotid vessel wall and subsequently denude the vessel endothelium (Fig 1). Across 4 separate batches of surgery (n = 12/each) the injury model was found to be a simple and quick procedure resulting in high animal survival rates. Surgical times lasted approximately 23 mins from animal knockdown to full pedal reflex recovery with an average survival rate of 91.7% (Table 1).

**Table 1:** Surgery records across 48 rat procedures performed in 4 separate batches.

Animals	Mean weight	Average surgical time	Survival rate
N = 48	297 ± 7 grams^a^	23 ± 3 mins^a^	91.7%

^a^± indicates SEM.

### Endothelial denudation

**Fig 2:**
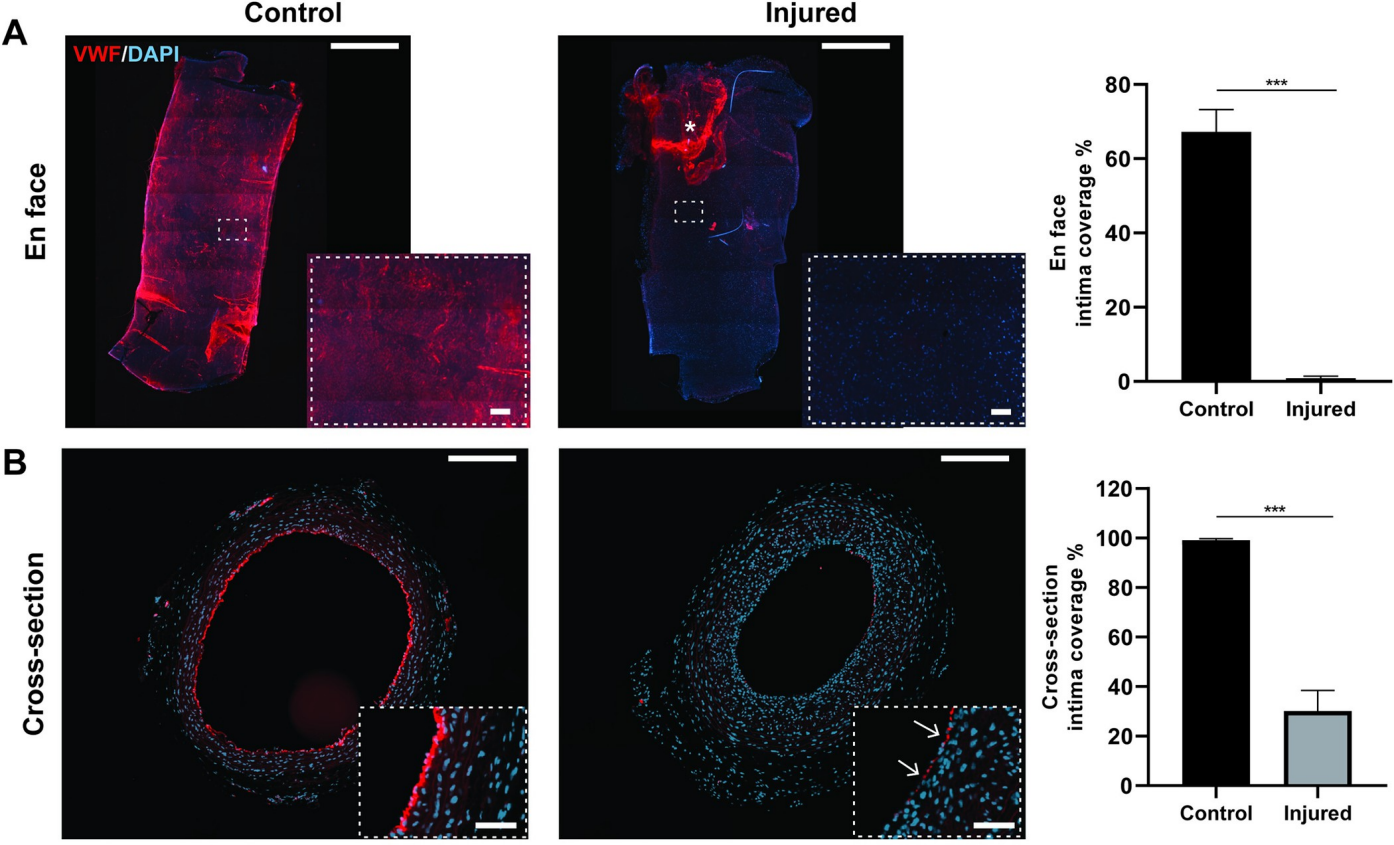
Microforcep and catheter injury causes near complete and sustained denudation of the vessel endothelium. A) En-face imaging of CD31^+^ stained carotid arteries and quantification of CD31^+^ coverage of DAPI counterstained vessels immediately following injury. Denuded endothelium folded into the distal side of the artery (*); n = 3 control, n = 3 injured B) Cross-sectional CD31^+^staining of injured vessels 2 weeks post-injury and quantification of CD31^+^ lumen coverage. Inset showing magnified endothelium with concentric staining in controls and incomplete staining in injured vessels (arrows); n = 4 control, n = 16 injured. Data presented as average ± SEM; scale bar = 0.5mm, inset = 100μm; ***p<0.01.

To determine the extent of endothelial denudation, vessels were explanted immediately after injury and 2 weeks post-injury. To visualise the injury to the endothelium en face- and cross-sections, respectively, were stained with the endothelial marker CD31. Enface staining showed an 87 ± 0.52% reduction of endothelial coverage across the length of the explanted vessel (Fig 2A) indicating a significant level of vessel denudation. Cross-sectional staining at 2 weeks post-injury was chosen over en-face staining to determine endothelial lumen coverage more accurately. Cross-sectional staining showed that the initial disruption to the endothelium was sustained for up to 2 weeks (Fig 2B), indicated by only a 30 ± 8% recovery in endothelial coverage over this period. This demonstrated that the injury was sufficient for significant endothelium denudation with a slow rate of native endothelial re-growth or vessel healing over 2 weeks.

### SMC differentiation & hyperplasia

**Fig 3:**
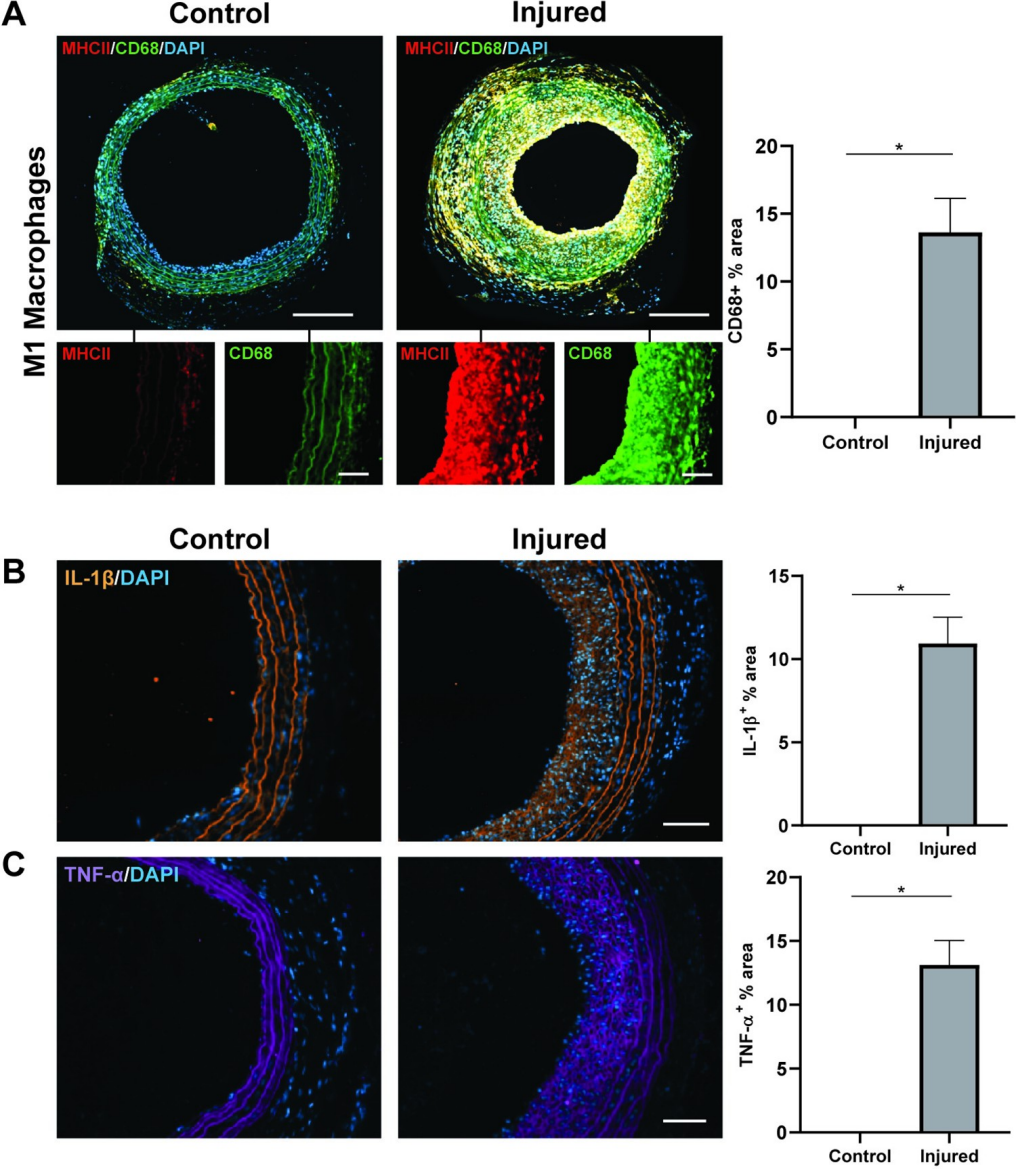
Development of neointimal hyperplasia and smooth muscle cell phenotype switching at 2 weeks post-injury. A) Representative photos of H&E staining depicting neointimal area and SMC-α, PCNA, and DAPI triple immunostaining showing switch of SMC phenotype in control vessels versus injured vessels; solid line indicates the original lumen and dashed line shows the lining of the neointima; n = 3 control, n = 11 injured B) Quantification of H&E staining showing vessel occlusion percentage and C) immunostaining showing total SMC-α^+^ neointimal area and D) PCNA/SMC-α ratio indicating proportions of contractile and synthetic smooth muscle cell phenotypes in the neointima; n = 3 control, n = 11 injured. Data presented as average ± SEM; scale bar = 300μm, inset = 50μm; *p<0.05, **p<0.01.

The most reported metric of all vessel injury models is neointima formation arising from the migration and over-proliferation of SMCs from the vessel wall, driven by a contractile-to-synthetic phenotype switch. To validate whether our model activated a similar response, vessels were explanted two weeks post-injury and stained with H&E to evaluate vessel patency and separately co-stained with PCNA and SMC-α to determine the ratio of contractile and synthetic phenotypes (Fig 3A). Neointima formation was represented by a quantification of vessel occlusion. Compared to uninjured controls, our injury produced an average of 65 ± 11% vessel occlusion (Fig 3B). Within this developing neointima we found 24 ± 4% of the area was positively stained with SMC-α actin (Fig 3C). Within these smooth muscle cell fractions, we further observed a 76 ± 5% expression of the synthetic phenotype indicated by the PCNA/SMC-α ratio (Fig 5D). This validated that our injury-induced vessel occlusion was consistent with classical neointima formation responses involving SMC phenotype differentiation.

### Vascular inflammation

**Fig 4:**
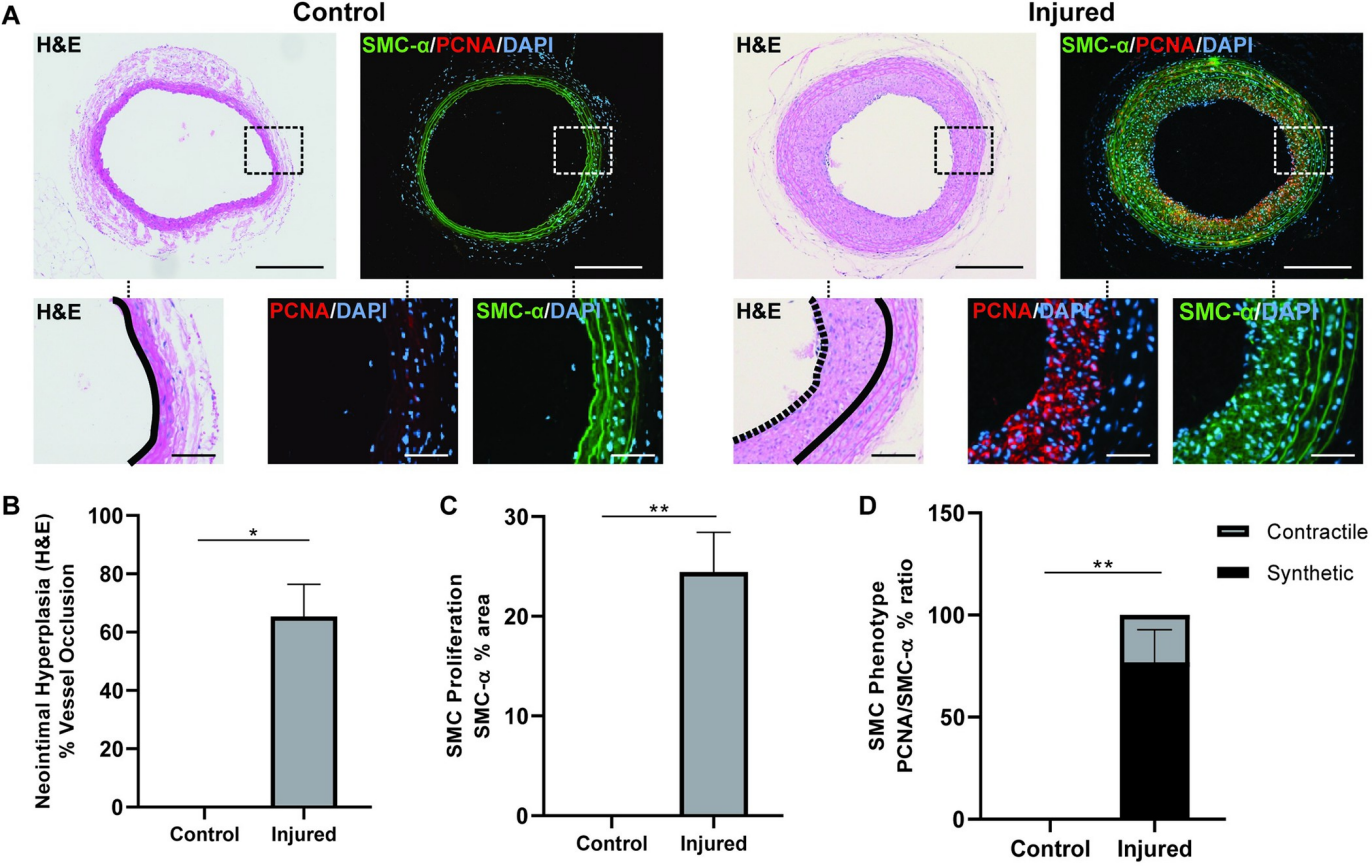
Activation of vascular inflammation at 2 weeks post-injury. A) Representative photos of MHC Class II (MHCII), CD68, and DAPI triple immunostains labelling M1 macrophages in control versus injured vessels. Co-localisation of MHCII and CD68 appear yellow in the merged images and indicate M1 macrophages. Insets show individual channels of MHCII (red) and CD68 (green). Quantification of total macrophages represented as total CD68 staining area as percentage of the vessel cross section; n = 3 control, n = 9 injured. Similar representative imaging and quantification of inflammatory cytokine expression in the neointima for B) IL-1β and C) TNF-α; n = 3 control, n = 9 injured. Data presented as average ± SEM; scale bar = 300μm, inset 50μm; *p<0.05.

To evaluate the levels of immune activation, vessels were next stained for total macrophages (CD68^+^) and those polarised to the pro-inflammatory (M1) phenotype, using the MHCII^+^ and iNOS^+^ marker. In injured vessels 2 week-post injury, CD68^+^ was observed both within the vessel wall and the neointima highlighting the spatial recruitment of macrophages to the vessel injury (Fig 4A). Positive co-staining iNOS (S4 Fig) further indicated that these macrophages were of the M1 phenotype. Quantification showed that the neointimal area consisted of 13 ± 2% total macrophages. Further evaluation of the immune response was assessed by quantifying levels of inflammatory cytokine expression. Expression levels of IL-1β and TNF-α showed 10 ± 1% and 13 ± 1% of the total neointimal area, respectively (Fig 4B and 4C). These results were demonstrative of vessel damage robust enough to trigger the classical immune responses consistent with NH development.

### Neointima remodelling

**Fig 5:**
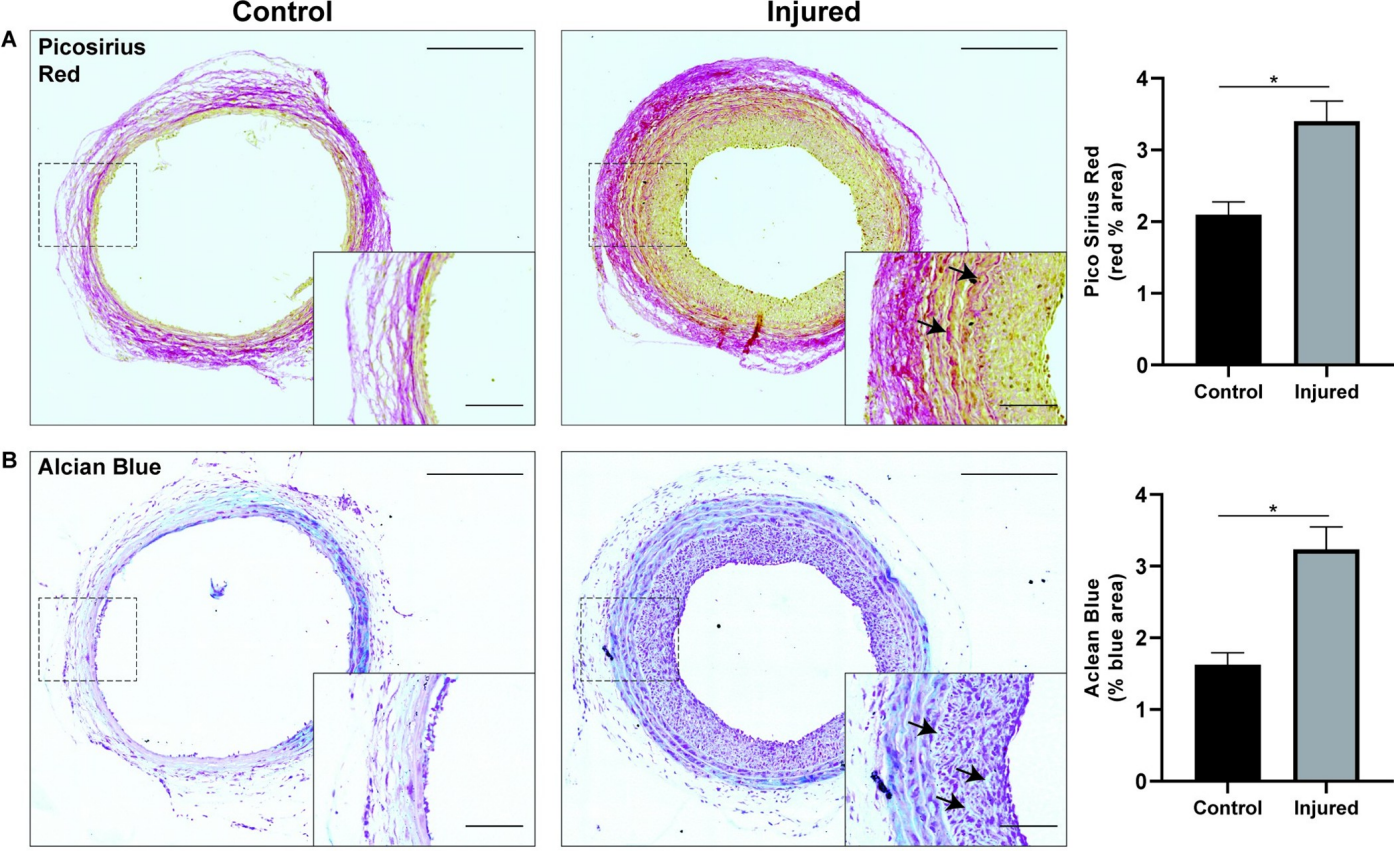
Neointimal remodelling at 2 weeks post-injury. A) Representative photos of collagen deposition (red) using picrosirius red (PSR) and quantification represented as a percentage of the neointimal area stained red (arrows); n = 3 control, n = 10 injured B) Representative photos and quantification of proteoglycans (blue) using Alcian Blue and quantification represented as a percentage of the neointimal area stained blue (arrows); n = 3 control, n = 13 injured. Data presented as average ± SEM; scale bar = 300μm, inset = 50μm; *p<0.05.

Matrix deposition within the occlusive neointima is critical to the pathogenesis of atherosclerosis making it a beneficial feature of *in vivo* models of vessel disease. To determine whether the hyperplasia induced by our model could recapitulate matrix deposition consistent with clinically observed pathology, Pico Sirius Red (PSR) and Alcian Blue (AB) stains were used to visualise collagen and proteoglycan deposition, respectively. Collagen was found to be significantly upregulated within both the vessel wall and neointima, showing an overall total vessel increases of 157% (Fig 5A). Similarly, proteoglycan synthesis was found to be elevated in both the forming hyperplasia and vessel wall, calculated at a total increase of 188% within the injured vessel (Fig 5B).

## Discussion

Microforcep and catheter-induced vessel injury is a simple and quick surgical procedure that can be effectively used to model the characteristic biology of NH development. The procedure involves mechanical vessel distension by expanding a microforcep tool followed by complete vessel denudation using a conventional needle catheter, both of which are inserted through an arteriotomy of a rat common carotid artery. A strong advantage of our model is that it does not require permanent ligation of distal blood vessels past the internal-external carotid bifurcation (i.e. superior thyroid, occipital, and ascending pharyngeal). Using common surgical instruments readily available to any small animal surgery lab rather than custom-designed balloon catheters that require longer vessel lengths to reproducibly induce injury, our method enables a similar injury over half the required vessel length of rat balloon injury. Similar to the balloon injury, vessel distention and endothelial denudation can occur objectively between operators as the microforceps have maximum opening widths of 1.5mm and needle catheters are standardised to 22-gauge diameters. This combines into a single procedure the two modes of injury that have made mouse wire and rat balloon injury models a highly consistent means of evaluating new restenosis therapies.

Our results firstly show that the model produces near complete endothelial denudation immediately after injury with substantial disruption of the endothelium sustained for at least 2 weeks with only approximately 30% recovery of the endothelium. This is more comparable to other balloon injury models in rats with reports of 15% recovery [22]. Relative to other models including the mouse wire injury which has reported up to 75% endothelial recovery by 7 days [23], endothelial recovery in our model progresses significantly slower. Even compared to larger animal injury models such as rabbit carotid and iliac balloon injury which report near complete re-endothelization in as little as 14 days [24], our model is closer to clinical observations of slow recovery where complete re-endothelialisation can extend even beyond one year after balloon injury in patients [25]. These reduced levels of re-endothelialization at 14 days have implications for better study and evaluation of endothelial-targeted therapies. Longer healing makes it easier to study the biology of endothelial recovery, allowing for a larger number of timepoints to sample from and providing potentially more accurate examination of mechanisms with increased temporal resolution.

The major reported metric of balloon injury models is neointimal growth and luminal patency, in our model we observed an average of 65% luminal occlusion. To make comparisons to neointimal areas which are often reported in mm^2^ throughout the literature, we back calculated our occlusion percentages to an average 0.228 ± 0.02 mm^2^ neointimal area after 2 weeks of injury. Compared to balloon injury models, which have reported neointimal areas of 0.255 ± 0.04 mm^2^ [26], 0.218 ± 0.014 mm^2^ [22] and 0.21 ± 0.03 mm^2^ [27], our injury yielded similar levels of neointimal growth. To the best of our knowledge, the only model with higher relative levels of NH was in a mouse model of ligation injury where the occlusion percentage of 80% was reported [28]. Previous studies have shown the extent of vascular damage is directly proportional to neointima formation [26]. This suggests that the level of our injury is comparable to rat balloon models, replicating the similar features of the rat balloon model which have made it an invaluable tool for the evaluation a wide range of neointimal reduction strategies.

Developing new therapeutic approaches to restenosis relies heavily on a solid understanding of neointimal development and its biological underpinnings. Hyperplasia arising within our model was consistent with high percentages of synthetic SMCs phenotype switching reflective of an unstable and growing neointima. Here we have used the proliferative marker PCNA to indicate synthetic SMC phenotypes, which allows us to determine the state of neointimal hyperplasia development. High levels of PCNA staining will indicate that the SMCs in the neointima are continuing to proliferate and therefore hyperplasia is likely to increase in size. Other studies have also used Ki67 as a proliferative marker as well as the apoptotic marker Caspase 3. The markers of choice reported in each study are largely dependent on the mechanism of action for therapy being evaluated. For example, proliferative markers, PCNA and Ki67, were appropriate for anti-proliferative drugs such as sirolimus which directly target the mTOR pathways [29]. Cytotoxic drugs such as paclitaxel would benefit with apoptotic markers such as caspase 3 [30]. Regardless of the distinct markers used, the reproducibly high levels of synthetic SMC switching in our model would enable the evaluation of similar anti-proliferative approaches and/or mechanistic studies into contributions of phenotype switching in neointima development.

In addition to the crucial role of smooth muscle cells, mounting evidence points to the important role of vascular inflammation in the development of NH [31]. Our injury model elicits a highly significant immune response consistent with increased macrophage recruitment and concomitant pro-inflammatory cytokine secretion as previously reported in both the rat balloon and mouse wire injury models [32]. Further analysis into macrophage M1 activation showed that the phenotype could be positively and robustly identified using the MHC Class II cell surface marker. This analysis feature of the model may be beneficial to the evaluation of anti-inflammatory approaches which target macrophage polarisation to mitigate NH development. This is consistent with recent rat balloon injury studies which have shown broad identification using the CD68 marker as an indicator of macrophage infiltration, an event critical to the development of neointimal hyperplasia [33,34]. This macrophage behaviour is closely tied to pro-inflammatory cytokine profiles which include elevated levels of IL-1β and TNF-α. Achieving sustained levels of these cytokines in our model is suggestive of the injury being able to drive a comprehensive inflammatory microenvironment which is consistent with balloon and wire injuries. These collective responses have implications for the evaluation of all anti-inflammatory approaches which are becoming an increasing focus of anti-restenosis therapies. Comparative analysis of macrophage behaviour between treatment groups may allow for an improved predictive outcome of neointima hyperplasia progression.

Lastly, injured vessels within our model showed increased collagen deposition and proteoglycan synthesis both within the vessel wall and the developing neointima. While these are not commonly reported metrics in rodent-based injury models, both are critical vessel remodelling events that serve as precursors to more significant downstream vascular pathologies. In humans both balloon angioplasty and stenting are known to cause collagen accumulation in the arterial intima and media/adventitia layers. The processes underlying this occurrence are still not fully understood however, and numerous studies have acknowledged that both intact fibrillar collagen and its degradation are both integral to vascular repair [35]. Similarly, the presence of proteoglycans are naturally low in the ECM of vascular tissue, but increases dramatically in all phases of vascular disease. Proteoglycans have been repeatedly shown to accumulate in vascular lesions of both humans and in animal models in areas of the vasculature that highly susceptible to atherosclerotic development (i.e. branch points). Circulating lipids are commonly found to associate with proteoglycan deposits and establish the beginnings of plaque development [36]. This has inspired new research efforts focused on targeting proteoglycan synthesis and interactions in vascular remodelling [37,38].

## Conclusion

Using a surgical technique that provides mechanical distention and endothelial denudation injury in two consecutive procedures, our new vascular injury model was able to reproduce the main outcomes of vascular injury, including neointimal hyperplasia and re-endothelialization, at levels comparable to the current established models of restenosis. This method involved a simple modification to current injury models yet may offers complementary improvements, most notably the access to the larger diameter rat vessels, shorter vessel lengths required for injury, and the lack of permanent ligation/disruption of distal blood vessels. This feature offers a main advantage as injury is only induced locally without permanent ligation or disruption of blood flow in collateral vessels. This could perhaps supplement restenosis injury research by providing a closer representation of the physiological response to vascular damage following endovascular injury. Despite the model’s procedural modifications, the traditional vascular damage responses, such as increased M1 macrophage activation, pro-inflammatory cytokine release, SMC contractile-to-synthetic differentiation, and proteoglycan and collagen deposition in the developing neointima are additional features of the model which can be accurately quantified and demonstrate remarkable consistency over 2 weeks across multiple batches of surgeries. The inclusion of this method into the current suite of rodent-based arterial injury models may help facilitate more in-depth analysis of pathogenic causes of restenosis and accurate evaluation of future anti-restenosis therapies.

## Supporting information

S1 Fig Surgical equipment and setup for performing microforcep and rat carotid injury. 1) Surgical microscope 2) vessel clamps and 22G needle catheter 3) No.22 scalpel blade 4) sterilised gauze 5) surgical forceps and 6) microscissors. 7) Microforceps for performing vessel distention 8) sterilised gauze tips 9) 9–0 silk suture for arteriotomy closure and 10) 3–0 silk suture to close up neck incision.
(TIF)

S2 Fig Schematic diagram of carotid artery surgery. A 1.5 cm segment of the carotid artery is isolated just proximal to the bifurcation of the internal and external carotid artery using vessel clamps. At the most proximal vessel clamp, an arteriotomy is performed to create an opening for the insertion of microforceps and needle catheter.
(TIF)

S3 Fig Schematic diagram of histopathology and immunohistochemistry staining analysis. Explanted common carotid arterial segments were sectioned along their length in 5μm sections. Three sections were placed on each slide, and for each stain, three equidistant slides were selected at the proximal, middle, and distal ends. These slides were averaged to create a single data point for the sample. The total samples were then averaged and presented with ± as the standard error of the mean (SEM).
(TIF)

S4 Fig Neointimal hyperplasia immunostaining of M1 macrophages. A) Representative co-staining using iNOS (M1) and CD68 (macrophages), scalebar = 300μm; inset: individual channels of iNOS and CD68, scalebar = 100μm. B-C) Secondary antibody (no primary) control images of AlexaFluor488 (AF488—green) and AlexaFluor594 (AF594) secondary antibodies showing negative staining in the neointima but auto-fluorescence of elastic laminae, scalebar = 100μm.
(TIF)

S1 File Raw Data. PDF file containing all raw data presented throughout the manuscript.
(PDF)
