# Supplementary figures and images for: Highly reproducible rat arterial injury model of neointimal hyperplasia
Source: PLoS One. 2023 Aug 17;18(8):e0290342. doi: 10.1371/journal.pone.0290342 (PMC10434902; doi:10.1371/journal.pone.0290342)

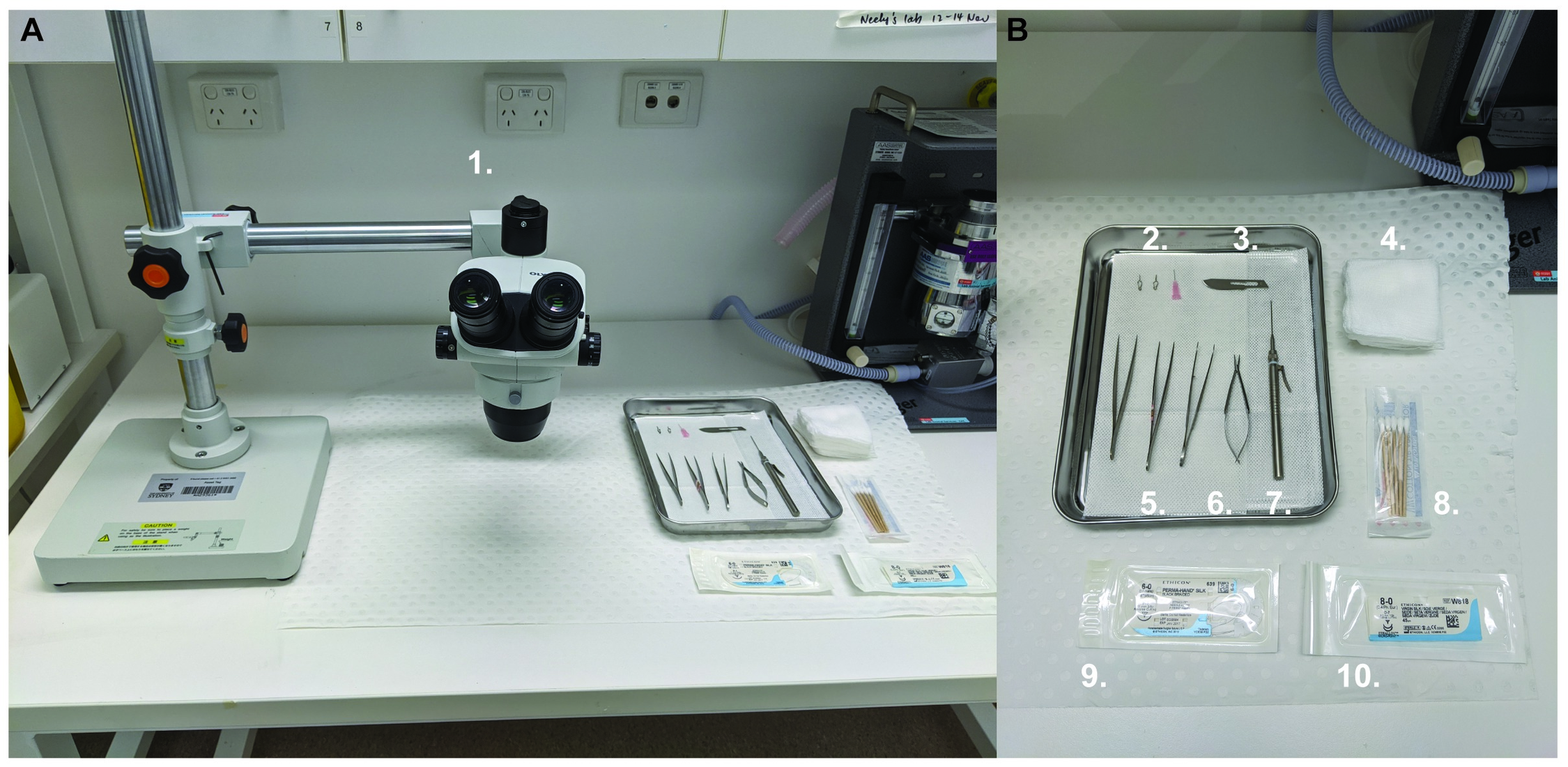

Supplement: S1 Fig — 1) Surgical microscope 2) vessel clamps and 22G needle catheter 3) No.22 scalpel blade 4) sterilised gauze 5) surgical forceps and 6) microscissors. 7) Microforceps for performing vessel distention 8) sterilised gauze tips 9) 9–0 silk suture for arteriotomy closure and 10) 3–0 silk suture to close up neck incision. (TIF) [file pone.0290342.s001.tif]

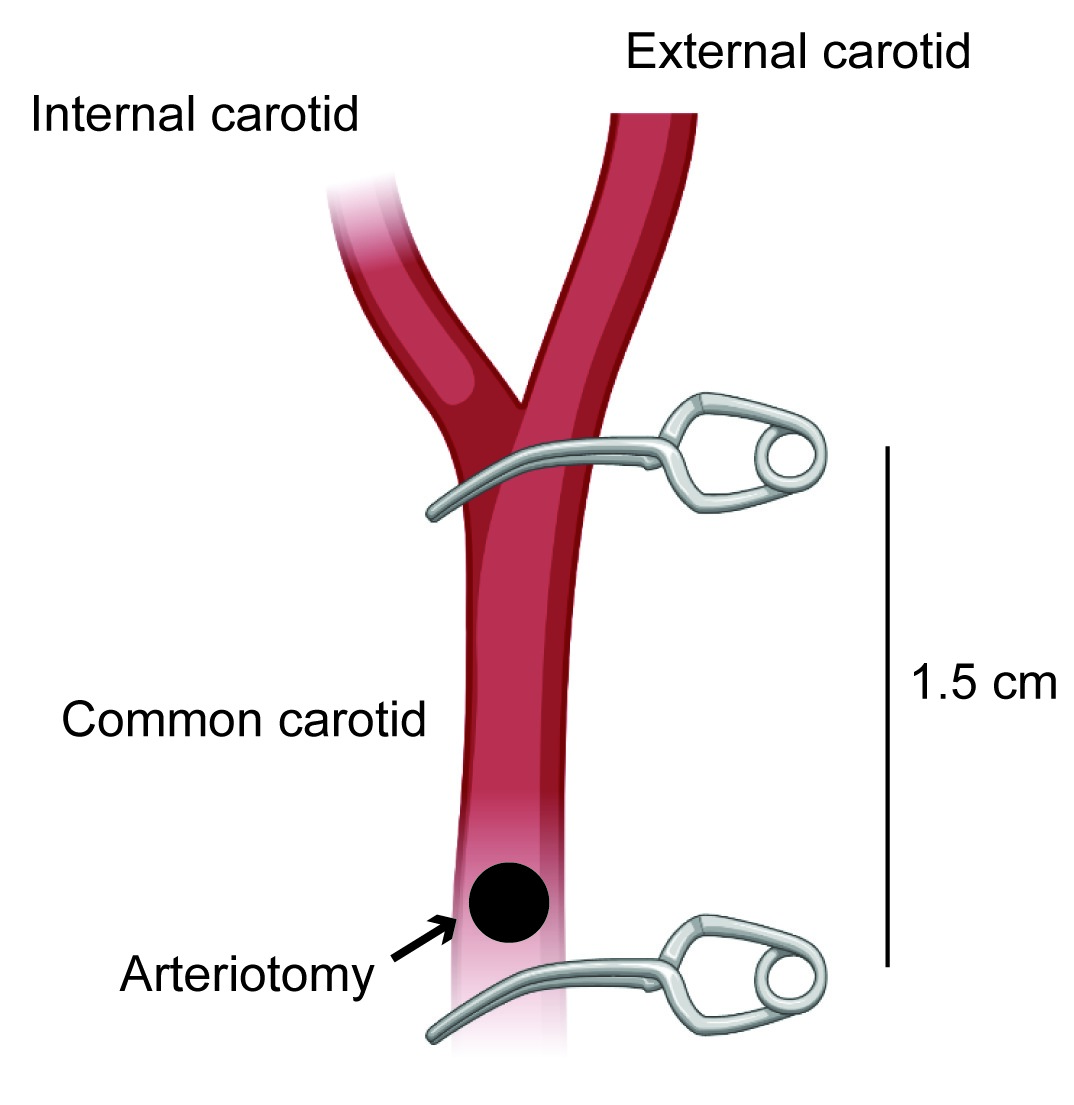

Supplement: S2 Fig — A 1.5 cm segment of the carotid artery is isolated just proximal to the bifurcation of the internal and external carotid artery using vessel clamps. At the most proximal vessel clamp, an arteriotomy is performed to create an opening for the insertion of microforceps and needle catheter. (TIF) [file pone.0290342.s002.tif]

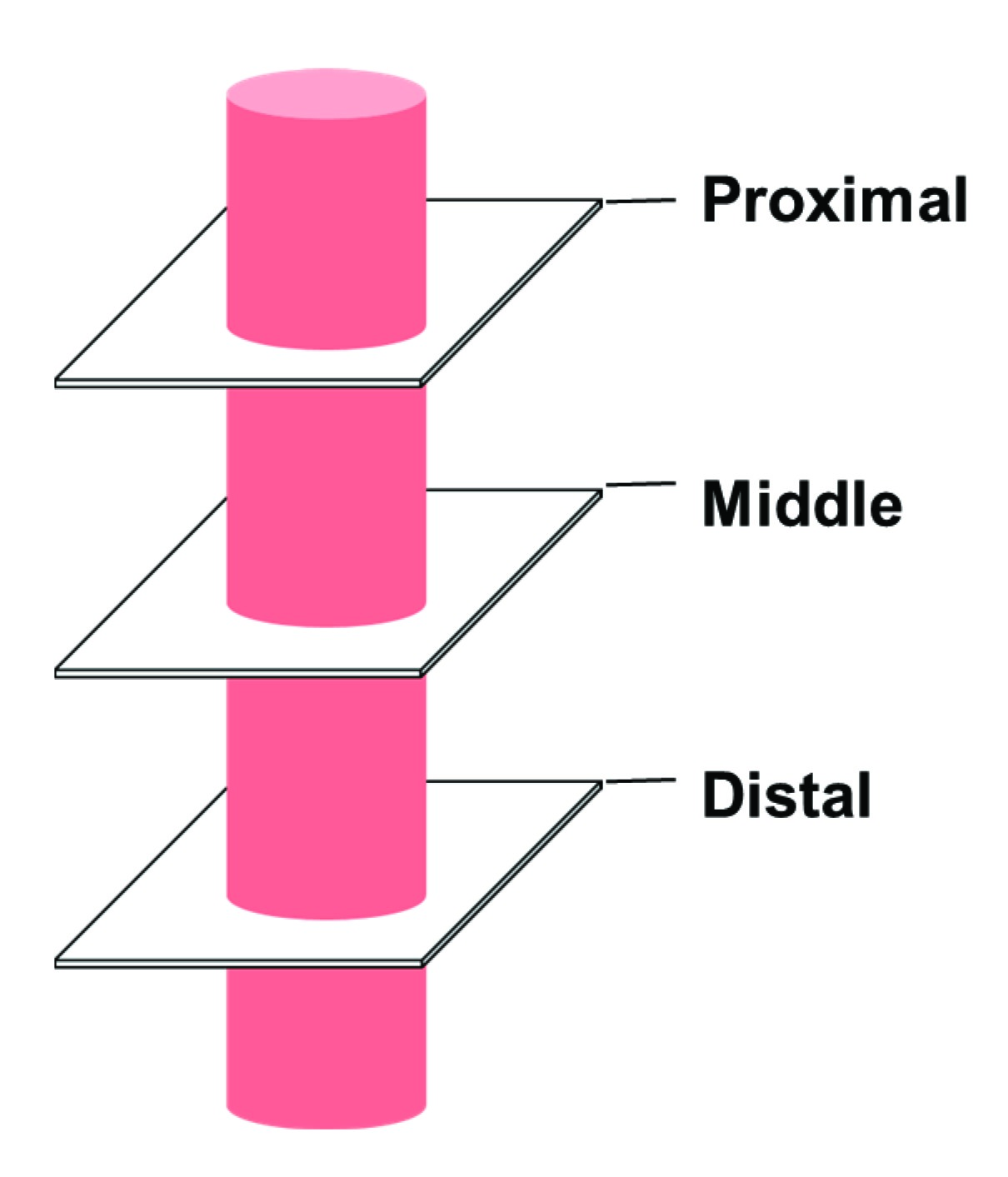

Supplement: S3 Fig — Explanted common carotid arterial segments were sectioned along their length in 5μm sections. Three sections were placed on each slide, and for each stain, three equidistant slides were selected at the proximal, middle, and distal ends. These slides were averaged to create a single data point for the sample. The total samples were then averaged and presented with ± as the standard error of the mean (SEM). (TIF) [file pone.0290342.s003.tif]

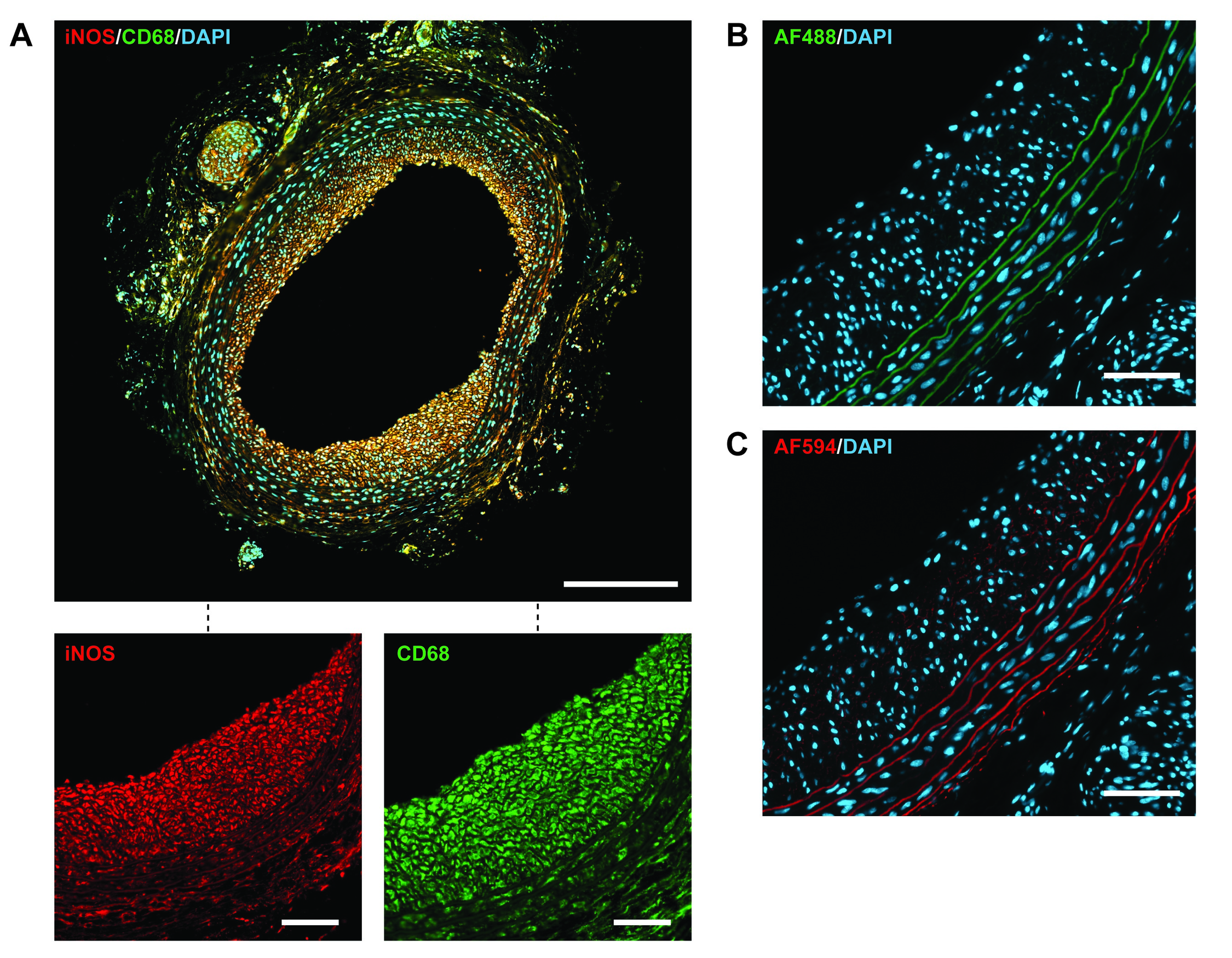

Supplement: S4 Fig — A) Representative co-staining using iNOS (M1) and CD68 (macrophages), scalebar = 300μm; inset: individual channels of iNOS and CD68, scalebar = 100μm. B-C) Secondary antibody (no primary) control images of AlexaFluor488 (AF488—green) and AlexaFluor594 (AF594) secondary antibodies showing negative staining in the neointima but auto-fluorescence of elastic laminae, scalebar = 100μm. (TIF) [file pone.0290342.s004.tif]
