# Supplementary material for: Highly reproducible rat arterial injury model of neointimal hyperplasia
Source: PLoS One. 2023 Aug 17;18(8):e0290342. doi: 10.1371/journal.pone.0290342 (PMC10434902; doi:10.1371/journal.pone.0290342)
Supplement: S1 File — PDF file containing all raw data presented throughout the manuscript. (PDF) [file pone.0290342.s005.pdf]

CD31 % coverage

Control	Injured
66.38792	1.837096
78.0079	0.789107
57.39078	0.018275

CD31 % coverage

Control	Injured
97.38089	0
99.16806	0
100	8.226667
100	0
	11.66667
	0
	58.56667
	95.46667
	73.16667
	44.33333
	63.66667
	13.33333
	25.33333
	79.33333
	9
	0

Control	Injured
0	100
0	97.29503
0	32.85493
	96.47821
	57.25827
	100
	22.11649
	71.21259
	0
	41.65195
	100

Control	Injured
0	35.8
0	51.7
0	4
	23.4
	28
	25.1
	10.6
	27
	12
	31.2
	20.1

Control	Injured
0	55.02793
0	53.77176
0	80
	90.17094
	98.57143
	87.251
	89.62264
	82.22222
	70
	54.80769
	83.58209

Control	Injured
0	22.45
0	10.153
0	11.756
	6.06
	6.67
	20.69
	9.753
	26.409
	8.744

Control	Injured
0	11.07
0	4.682
0	1.494
	12.048
	8.748
	18.5
	17.423
	14.314
	12.543
	4.478
	17.166
	8.668

Control	Injured
0	9.506
0	6.968
0	19.449
	19.101
	13.491
	15.159
	8.206

Control	Injured
1.92	5.515
1.925	3.031
2.449	2.876
	2.681
	2.963
	3.493
	3.388
	3.299
	2.519
	4.248

1.582	5.54
1.936	3.566
1.353	4.442
	4.399
	3.908
	2.378
	2.228
	2.148
	2.403
	2.584
	2.332
	4.025
	2.105
